# Treatment strategy for late‐life depression

**DOI:** 10.1002/pcn5.91

**Published:** 2023-05-07

**Authors:** Hajime Baba

**Affiliations:** ^1^ Department of Psychiatry, Faculty of Medicine, Juntendo Koshigaya Hospital Juntendo University Saitama Japan; ^2^ Department of Psychiatry & Behavioral Science Juntendo University Graduate School of Medicine Tokyo Japan

**Keywords:** antidepressant, depression, elderly, late‐life, treatment

## Abstract

With the unprecedented aging of the world's population, the number of elderly patients with depression is expected to increase. However, management and treatment of late‐life depression (LLD) is more difficult than in early adults. Prior to treatment, diagnosis must take into account the differentiation from, and comorbidity with, organic brain diseases such as dementia and delirium, as well as depression caused by other physical diseases or medications. As clinical features of LLD, treatment response tends to be poor in older patients and recurrence rates are higher than those in early adult patients, therefore psycho‐social interventions on the basis of the patient's background and condition are important for LLD. The first‐line treatment strategy generally depends on the severity of the depression. Systematic psychotherapies, including cognitive behavioral therapy and problem‐solving therapy, have been reported to reduce depressive symptoms in LLD. Regarding pharmacotherapy, newer antidepressants are recommended for LLD, but careful attention to adverse events is required. Treatment using neuromodulation is also reported to be useful for LLD. In the current review, for further‐line treatment, treatment strategies were divided according to the level of first‐line treatment response. Evidence indicates that LLD is more heterogeneous than depression in younger adults, therefore when treating LLD patients it is necessary to take various conditions and situations into consideration, and to provide detailed treatment that is tailored to each patient.

## Graphical abstract

This review describes treatment strategies with a particular focus on pharmacological treatments for late‐life depression (LLD) using recent guidelines, evidence from various studies, and the author's opinions as a clinical expert on LLD.

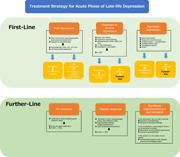

## INTRODUCTION

In 1910, Emil Kraepelin stated that “the area of presenile psychosis is probably the most unknown area of psychiatry at present” in the eighth edition of the textbook *Presenile Psychosis*. Over 100 years later, psychiatric problems exhibited among elderly people, particularly depression, are extremely diverse and probably more complex than those at the time of Kraepelin's writing.^1^ With the unprecedented aging of the world's population,^2^ the number of elderly patients with depression is increasing, and it is not unusual for elderly patients with depression to seek help in primary care settings. However, management and treatment in late‐life depression (LLD) is more difficult compared with that in early adults.

Elderly patients with depression often resemble those with dementia because they show inhibition of thought, and reduced attention and concentration (pseudodementia), therefore it is often difficult to differentiate LLD and dementia. Moreover, studies indicate that LLD is a risk factor for dementia, suggesting that it is not unusual for patients to transition from LLD to dementia. In addition, delirium, drug‐induced depression, and depressive symptoms caused by physical disease are also often observed in elderly people. Prior to treatment, differentiation from and comorbidity with organic brain diseases such as dementia, delirium, and depression caused by physical disease or medications must be taken into consideration.

Regarding treatment, a number of studies have reported that there is little difference in the effects of pharmacotherapy between elderly and early adult patients with depression.^3^ However, recent studies have shown that the treatment response tends to be poor in older patients, and the recurrence rate is higher than that in early adult patients,^3^ therefore psychosocial interventions that are based on the patient's background and condition are important for LLD. Elderly patients often face a variety of loss experiences, and more evidence regarding the effectiveness of adequate psychotherapeutic interventions is required. Recent studies have reported evidence that systematic psychotherapies can reduce depressive symptoms in LLD, including cognitive behavioral therapy (CBT) and problem‐solving therapy (PST).^3^ Treatment using neuromodulation is also reported to be useful for LLD.^3^

The Japan Society for Mood Disorders issued the Guidelines for the Treatment of Depression in the Elderly^1^ in July 2020, and the first revision was published in 2022 as the Guidelines for diagnosis and treatment of depression in older adults: A report from the Japanese Society of Mood Disorders.^3^ The current review describes treatment strategies with a particular focus on pharmacological treatments for LLD using these guidelines, evidence from various studies, and the author's opinions as a clinical expert on LLD.

## CONSIDERATIONS BEFORE THE INTRODUCTION OF TREATMENT

### Differential diagnosis and factors affecting LLD

Although differentiation between LLD and dementia is suggested as a clinical challenge, confirmation of depressive symptoms is clinically important whether or not dementia is present. It has been reported that over 50% of patients with dementia have depressive symptoms,^4^, ^5^, ^6^ and these symptoms should be treated. Differentiation between depression and apathy is clinically important. Loss or reduction of motivation is a core feature of apathy, and the symptoms of apathy can be grouped into three categories: emotional impairments (e.g., flattening of emotions and emotional blunting), cognitive impairments (e.g., indifference), and behavioral impairments (e.g., lack of initiation).^3^, ^7^ Apathy appears in patients with frontal lobe injury, vascular disease, and other neurodegenerative disorders. Although depression and apathy are sometimes comorbid, opposite approaches are required (i.e., patients with depression need rest, whereas patients exhibiting apathy need activation). The points of clinical differentiation for depression and apathy are shown in Table 1.^3^

**Table 1:** Points of clinical differentiation for depression and apathy.^3^

Depressed state	Apathy
Emotions and affect	
Depressive mood	Flattening of emotions, emotional blunting
Depression, grief, anxiety, irritation, and hopelessness	Diminished or lack of emotional response to any event
Interest	
Loss of interest and pleasure	Indifference
Interest in negative events and poor self‐condition is rather excessive (e.g., hypochondria)	Loss of interest in both positive and negative events Loss of interest in self‐condition
Motivation and activity	
Psychomotor inhibition (retardation)	Lack of initiative
Retained motivation to act	Lack of motivation to act
Reduced activity with conflict and distress	Reduced activity without conflict or distress

It may sometimes be necessary to differentiate depression and delirium, particularly hypoactive delirium. Compared with depression, delirium has an acute onset and fluctuating symptoms, and is characterized by very scattered attention and concentration, confused speech, and disorientation.^3^, ^8^

Many elderly people have physical and/or organic brain diseases, and drugs are often administered to treat these conditions, therefore the presence of depression caused by these disorders and drugs should be checked.

### Evaluation of the condition and basic intervention

Depressive symptoms and physical conditions must be evaluated in patients with LLD. Suicidal ideation/attempt, appetite/feeding, and general condition are particularly important considerations in decisions about admission and modified‐electroconvulsive therapy (m‐ECT). The Geriatric Depression Scale is a self‐rating scale developed to screen for LLD.^3^, ^9^

As basic interventions, psychoeducation and environmental adjustment should be implemented for the patient, family, and caregivers.^3^ It is important to show a receptive and empathic attitude that is considered against the background of various loss experiences (i.e., elderly people may experience deterioration in several areas, such as physical function, reduction in social roles, and bereavement of close relatives, in a relatively short period of time). In addition, regardless of whether the patient lives with family or alone, family members often experience conflicts between expectations of dignified attitudes to and disappointment with the patient, and may already be exhausted from caregiving. It is necessary to encourage understanding of late‐life mental health while also showing an empathic attitude toward patients' family members. LLD is associated with a high risk of suicide and should be treated carefully. If suicidal ideation or pessimistic behavior is observed, immediate and appropriate measures should be taken.

## FIRST‐LINE TREATMENT

**Figure 1:**
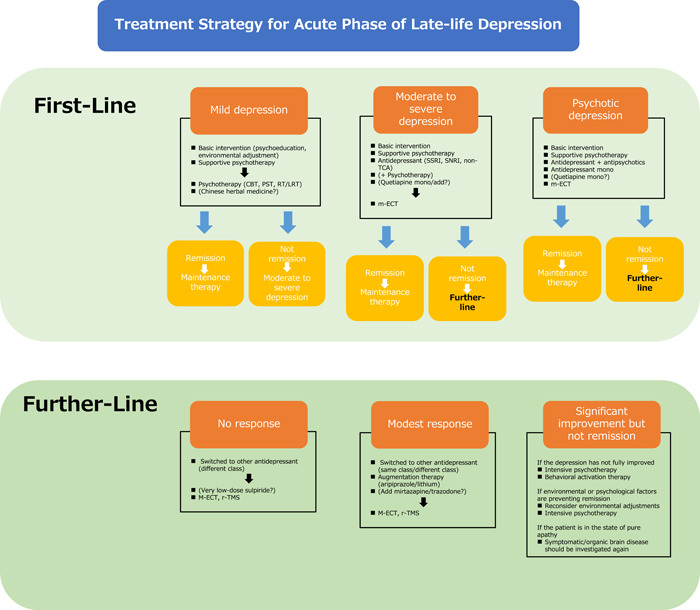
Treatment strategy for acute phase of late‐life depression. CBT, cognitive behavioral therapy; LRT, life review therapy; m‐ECT, modified‐electroconvulsive therapy; PST, problem‐solving therapy; RT, reminiscence therapy; SNRI, serotonin and norepinephrine reuptake inhibitor; SSRI, selective serotonin reuptake inhibitor; TCA, tricyclic antidepressant.

Treatment strategies for the acute phase of LLD are shown in Figure 1.

### Mild depression

Patients with LLD of mild severity often show improvement and remission with basic interventions such as psychoeducation and environmental adjustment, and supportive psychotherapy. Although the evidence regarding the efficacy of supportive psychotherapy is currently insufficient, all patients should receive this therapy.^3^ Evidence for the efficacy of CBT, PST, reminiscence therapy (RM)/life review therapy (LRT), and behavioral activation therapy have been reported for LLD.^3^ However, psychotherapy involves a burden for patients, and should be administered to patients with symptoms of mild to moderate severity. RM and LRT are commonly used with elderly patients. In this approach, reminiscence therapists adopt an accepting, empathic attitude to assist patients in the process of looking back on their lives and actively evaluating them. The aim of this approach is to deal with unsolved problems from the past, improve self‐esteem, and integrate the personality.^3^ Reminiscence therapy has also been shown to be effective in improving depressive symptoms in patients with dementia.^10^ Additionally, although the evidence is insufficient, clinical experience suggests that Chinese herbal medicine may be useful for patients with mild depression.

### Moderate to severe depression

Patients with symptoms of mild severity but who do not show improvement with basic intervention and psychotherapy, including supportive psychotherapy, and patients with moderate to severe depression should be provided with antidepressant pharmacotherapy. It has been reported that older age, baseline depression severity, baseline anxiety symptoms, current episode duration, physical illness, and executive dysfunction are negative predictors of treatment outcomes.^3^, ^11^ However, a meta‐analysis focused on LLD demonstrated that baseline severity was not associated with an antidepressant–placebo difference and placebo responses were large in the treatment of depressed elderly people,^12^ therefore how to induce placebo effects is an important factor in the success or failure of the treatment. To elicit a placebo effect, psychological approaches are needed that raise the expectations of treatment. For antidepressant treatment, guidelines recommend newer antidepressants, such as selective serotonin reuptake inhibitors, serotonin and norepinephrine reuptake inhibitors (SNRIs), and mirtazapine or nontricyclic antidepressants (TCAs) such as tetracyclic antidepressants and trazodone for LLD.^3^ In practice, antidepressants should be selected on the basis of the patient's symptoms and conditions, comorbid physical illness, concomitant medicines, and past treatment history. Japanese experts tend to prefer to use mirtazapine, sertraline, and escitalopram for elderly patients with depression.^13^ Although there is insufficient evidence regarding the selection of antidepressants on the basis of symptoms of depression, Japanese experts tend to select mirtazapine for patients who exhibit agitation and irritation, insomnia, loss of appetite, and suicidal ideation, escitalopram for patients who exhibit anxiety, and SNRIs for patients who exhibit loss of interest.^13^ These trends in antidepressant selection by Japanese experts may also apply to LLD. A randomized, double‐blind, placebo‐controlled study of extended release quetiapine monotherapy for LLD demonstrated significant superiority compared with placebo for quetiapine in response rate and remission rate.^14^ Moreover, significant improvements of depressive symptoms (Montgomery‐Åsberg depression rating scale total score, apparent and reported sadness, inner tension, reduced sleep, reduced appetite and pessimistic thought) by quetiapine were also seen at week 1. A recent network meta‐analysis of LLD showed that quetiapine efficacy was superior to that of most antidepressants.^15^ Although quetiapine was associated with significantly more dropouts because of adverse events compared with placebo, the mean risk ratio was not particularly high compared with antidepressants. On the basis of this evidence and clinical experience, quetiapine monotherapy may be a treatment of choice in the acute phase of LLD, especially in patients with agitation and inner tension. However, there is no evidence regarding the efficacy of quetiapine in preventing relapse/recurrence of depression or for its long‐term safety in LLD, therefore maintenance with quetiapine monotherapy is not recommended. The Japanese guidelines for LLD recommend that the efficacy of a minimal effective dose should first be confirmed when using antidepressants for LLD.^3^ However, because many newer antidepressants have shown efficacy and safety for elderly patients at the usual dose, it is recommended that when the minimal effective dose is insufficiently effective, it should be increased to the maximum dosage, while carefully monitoring adverse events.^3^

Severe depressive patients, especially cases in which suicidal ideation or pessimistic behavior is exhibited and cases in which the general condition has worsened because of appetite loss and dehydration, should be admitted and considered for treatment with m‐ECT. For patients with severe depression, TCAs may also be used in preference to newer antidepressants if they have been effective in the past. However, the use of TCAs should be considered more carefully in patients with poor general condition.

### Psychotic depression

Psychotic symptoms are frequently observed in LLD. On the basis of the evidence regarding the efficacy of quetiapine monotherapy for LLD mentioned above,^14^, ^15^ and the antidepressant actions as well as antipsychotic actions of quetiapine, clinical psychiatrists sometimes try quetiapine monotherapy for late‐life patients with psychotic features. However, evidence indicates that combination therapy with an antidepressant plus an antipsychotic is more effective than either treatment alone or placebo, and evidence is limited regarding treatment with an antidepressant alone or with an antipsychotic alone, including quetiapine.^16^ The latest National Institute for Health and Clinical Excellence (NICE) guidelines for depression in adults^17^ therefore recommend combination therapy with antidepressants and antipsychotics (e.g., olanzapine or quetiapine), and if patients do not wish to take antipsychotics in addition to antidepressants, an antidepressant alone is recommended. m‐ECT is a treatment choice for LLD patients with psychotic features who have not improved with medication.^3^ A meta‐analysis investigating the therapeutic effects of ECT for depression revealed that psychotic symptoms and older age were good predictors of remission and response.^18^

## FURTHER‐LINE TREATMENT

If treatment with first‐line antidepressants does not elicit a response, the Japanese guidelines for LLD recommend switching to other antidepressants rather than combining them with other antidepressants.^3^ Augmentation therapy with aripiprazole or lithium is also recommended in those guidelines.^3^ The choice of treatment strategy should be determined by shared decision making, considering the patient's condition, treatment history, and preferences.

If the current dose of antidepressants has not reached the maximum approved dose, an increase to the maximum dose should be attempted at least once if the medication is well tolerated. As mentioned in the section on first‐line treatment, many newer antidepressants have been reported to show efficacy and safety for elderly patients at the usual dose.^3^ It should be noted, however, that the efficacy of an antidepressant is not always dose‐dependent, and that increasing the dose may increase the risk of side effects.^17^

Most recent studies and guidelines define “response” as 50% improvement from baseline on depression scales, and treatment outcomes are also focused on response or no response. In actual clinical practice, however, the next treatment strategy often differs between patients exhibiting a complete lack of response to antidepressant medication, a slight improvement but not a 50% improvement, and a significant improvement but not remission.

### No response

Patients who show a complete lack of response to an antidepressant should be switched to a different antidepressant. Although the evidence for the benefits of switching to a different class of antidepressants is insufficient, such patients are generally switched to a different class of antidepressants. An antidepressant that has no effect is not appropriate as a base agent for augmentation therapy. If the patient does not respond at all to two antidepressants, first reconsider the diagnosis (e.g., apathy, hypoactive delirium, or another cause of symptomatic/drug‐induced depression‐like symptoms). If the diagnosis of depression is reconfirmed, then neuromodulation therapy such as m‐ECT or repetitive transcranial magnetic stimulation (r‐TMS) would be the modality of choice for treatment‐refractory depression. Before neuromodulation therapy, low doses of sulpiride are also worth trying. Although evidence is scant, many psychiatric clinicians have experienced the efficacy of sulpiride for LLD. Because this agent is a first‐generation antipsychotic and is prone to extrapyramidal symptoms, it should be used in very low doses (30–50 mg/day).

### Modest response

Patients who exhibit even a modest response to antidepressants can select two strategies. If the patient does not want to add medications, the treatment should be switched to another antidepressant. In this case, because the patient has responded to one antidepressant, switching to an antidepressant of the same class can also be expected to be effective. If the patient agrees to add another medication to augment the effects of the current antidepressant, augmentation therapy should be tried. Aripiprazole augmentation is the only strategy that has shown efficacy and a good tolerability profile for refractory LLD.^3^, ^19^ However, the lack of sufficient long‐term safety information and tardive adverse events such as dyskinesia should be taken into account. The level of evidence regarding the efficacy of lithium augmentation is lower than that for aripiprazole augmentation, and the risk of adverse effects is higher in late‐life patients because of poor renal function, comorbid physical diseases, and the taking of other medications. Although the NICE guidelines list the addition of different classes of antidepressants (e.g., mirtazapine or trazodone) as an option for depression in adults,^17^ the occurrence of adverse events should be noted for late‐life patients.

### Significant improvement but not remission

In cases of patients who exhibit significant improvement with first‐line treatment but do not reach remission, two factors should be considered: first, the possibility of residual depressive symptoms caused by inadequate treatment, and, second, the possibility of factors preventing remission. Although no clear distinction can be made, when depressive mood is clearly present, the depression may not be fully improved. If depressive mood is not prominent, but only anxiety remains, environmental or psychological factors may be involved. If depressive mood and anxiety are not prominent, and only loss of motivation persists, the patient may be in a state of apathy. Even if the depression has not fully improved, because pharmacotherapy has been significantly effective, the patient's nonpharmacological treatment should be intensified. In this state, the severity of depression is at a mild level, so somewhat more intensive psychotherapy and behavioral activation therapy can be attempted. If environmental or psychological factors are preventing remission, psychoeducation and environmental adjustments should be considered again. If psychological or social isolation or family conflict exists, social resources such as community circle activities or elderly day care may be helpful. If the patient is in a state of pure apathy, the possibility of organic brain disease should again be considered. An obsession with somatic symptoms may also be a symptom of organic brain changes, especially in the frontal lobes. Such patients should be considered for brain imaging, cognitive function testing, and neuropsychological testing to identify organic diseases, including dementia.

## MAINTENANCE THERAPY

The recurrence and relapse rates for depression are higher in elderly patients than in early adult patients.^3^ Because subsyndromal symptoms after remission are associated with a higher recurrence rate, sufficient maintenance therapy is important. One study reported that maintenance antidepressant therapy for 12 months prevented recurrence compared with a placebo (number needed to treat = 5), and that discontinuation owing to side effects did not lead to a difference.^20^ A meta‐analysis of three small randomized controlled trials showed that antidepressants can significantly reduce the recurrence rate for 1 year in patients who have achieved 6 months of remission.^21^ An expert consensus guideline for LLD^22^ recommends that the discontinuation of antidepressants should be considered 1 year after remission in those who have had a single episode of depression. Patients with two episodes of depression should continue antidepressants for 2 years. If the patient has had three or more episodes of depression, they should continue taking antidepressants for at least 3 years, or indefinitely. Predictive factors for recurrence include the severity of the last episode, the number of treatments needed to achieve remission, the number of years between depression episodes, and the presence of risk factors for depression, such as chronicity, disability related to medical multimorbidity, and a lack of social support.^22^ These factors also constitute important information for shared decision making for discontinuation of antidepressants. On the basis of these reports, Japanese guidelines for LLD recommend that continuation therapy should be maintained for at least 1 year after remission, and the duration of maintenance therapy should then be determined on the basis of the risk of recurrence and the preferences of the patient and their family.^3^ Although no evidence has been reported on this point, the author's personal observations suggest that patients tend to be at less risk of relapse/recurrence if they continue to visit their doctor after discontinuing pharmacological treatment, even if there are long intervals between visits.

## CONCLUSIONS

First, it is important for treatment strategies for LLD to carefully consider the differentiation between dementia, depression caused by physical and organic brain disease, and depression caused by drugs. However, it should be kept in mind that dementia and secondary depression can also be comorbid with primary depression.

Second, because elderly patients with depression may have been affected by psychological and environmental factors as well as organic brain factors, a psychosocial approach is important in the treatment of LLD. It is necessary to fully understand the clinical characteristics of LLD, understand the psychosocial background that affects the etiology and psychopathology of each patient, evaluate the patient's condition, and provide basic interventions on the basis of these factors.

Third, regarding specific treatments, psychotherapy, pharmacotherapy, and ECT/TMS have been shown to be useful for LLD. However, for pharmacotherapy, it is important to understand the effects of pharmacokinetics in elderly patients and to take extra care in monitoring the appearance of adverse events. In this review, Chinese herbal medicine, quetiapine monotherapy, and sulpiride administration at very low doses are also mentioned. However, the efficacy and safety of these treatments has not been fully confirmed, therefore when trying these treatments, patients should be carefully monitored. Moreover, further‐line treatment was divided into three strategies on the basis of response levels for first‐line treatment in this review. Although no evidence or guidelines are available for this division of strategies, it may be clinically useful.

Finally, LLD is more heterogeneous than depression in younger adults, therefore when treating late‐life patients it is necessary to take various conditions and situations into consideration and to provide detailed treatment that is tailored to each patient.

## AUTHOR CONTRIBUTIONS

The manuscript and figure were written and prepared by Hajime Baba.

## CONFLICT OF INTEREST STATEMENT

The author received grant funding from the Japan Society for the Promotion of Science and Esai, and speaker's honoraria from Otsuka Pharmaceutical, Sumitomo Dainippon Pharma, MSD, Meiji Seika Pharma, Eli Lilly, Yoshitomi Yakuhin, Janssen Pharmaceutical, Kyowa Pharmaceutical, Mitsubishi Tanabe Pharma, Ono Pharmaceutical, Pfizer, Esai, Takeda Pharmaceutical, Lundbeck, Mochida, Sawai, Kowa, EA Pharma, Mylan EPD, and Viatris.

## ETHICS APPROVAL STATEMENT

N/A.

## PATIENT CONSENT STATEMENT

N/A.

## CLINICAL TRIAL REGISTRATION

N/A.

## Data Availability

N/A.
